# Legacy samples in Finnish biobanks: social and legal issues related to the transfer of old sample collections into biobanks

**DOI:** 10.1007/s00439-019-02070-0

**Published:** 2019-10-16

**Authors:** Marjut Salokannel, Heta Tarkkala, Karoliina Snell

**Affiliations:** 1 University of Turku, Turku, Finland; 2 University of Helsinki, Helsinki, Finland

## Abstract

Biobank operations started officially in Finland in 2013 when the Biobank Act defining and regulating biobank operations came into force. Since then, ten biobanks have been established and they have started to collect new prospective samples with broad consent. The main corpus of biobank samples, however, consists of approximately 10 million “legacy samples”. These are old diagnostic or research samples that were transferred to biobanks in accordance with the Biobank Act. The focus of this article is on ambiguities concerning these legacy samples and their transfer in terms of legality, human rights, autonomy, and social sustainability. We analyse the Finnish biobank operations in the context of international regulation, such as the European Convention of Human Rights, the Oviedo Convention, European Charter of Fundamental Rights, the GDPR, and EU Clinical Trials Regulation, and show that the practice of using legacy samples is at times problematic in relation to this regulatory framework. We argue that the prevailing interpretations of these regulations as translated into the Finnish biobank practices undermine the autonomy of individuals by not giving individuals a right to consent or an actionable right to opt-out of the transfer of these legacy samples to the biobank. This is due to the fact that individuals are not given effective notification of such transfers. Thus, issues regarding the legal status of the biobank samples and the social sustainability of biobank operations remain a challenge for biobanks in Finland despite governmental efforts to create pioneering, comprehensive, and enabling legislation.

## Introduction

During the first decade of this millennium, biobank legislation was prepared in Finland. This legislation was expected to enable biomedical research and development through new prospective samples collected with consent. Finnish biobank legislation (Biobank Act 688/2012) entered into force in September 2013 and was celebrated as the first comprehensive modern biobank law to enable recontacting of donors and enhance the autonomy and privacy of individuals (Soini 2013; Tupasela 2015; MSAH 2016; Snell and Tarkkala 2019). Today, ten biobanks in Finland have been founded to meet the requirements of the law. In this way, Finland also defined biobanks through legislation—not all collections of specimens or collections of samples collected for research can be referred to as biobanks in terms of the law.

At the official governmental level and in the promotional material of Finnish biobanks, this regulatory landscape has been presented as an advantage for Finnish biobank operations in attracting interest and investments (e.g., Soini 2016; Ministry of Social Affairs and Health 2016: 5, 8; Biobanking in Finland 2018). Moreover, there is also an ongoing effort to build legislation for the health care sector to strengthen its potential for wider innovation purposes (see, for example, Ministry of Employment and Economy (MEE) 2016; Tarkkala et al. 2019). The recent law on secondary uses of social and health data (552/2019) as well as the planned genome centre (Ministry of Social Affairs and Health [MSAH] 2015) are part of this development. The effort to develop a robust and enabling legislative and regulatory environment has been used in strategy materials and innovation policy to underline Finnish competitiveness in biomedical research and development (Tarkkala et al. 2019). That is, it is expected that biobank operations will not only foster personalized medicine, but also research and development activities and innovations in biomedicine (Tarkkala 2019). Thus, for example, biobank collections can be utilized not only in research by universities, but also in different collaborations between academia and commercial partners as well as solely for, e.g., product development by companies. However, the Finnish biobank legislation has been under ongoing revision almost from the day that it was put into force.

In practice, the current legislation and its different interpretations in relation to actual biobank practices have created an equivocal situation. The broad informed consent model for prospective samples was regarded as justified, because it was seen to merit both research and the autonomy of individuals, as outlined in the Government proposal for the Biobank Act (HE 86/2011). This followed a general European and international trend to regard broad consent as a valid and preferred model for biobanking (Master et al. 2012). However, at the same time, when it became possible to start the collection of prospective samples, a transfer of the existing diagnostic sample collections in public hospitals, research collections in universities, and the epidemiological research collections of the National Institute of Health and Welfare (THL) into biobanks became possible without informed consent. It is the ambiguities concerning these older legacy samples and their transfer—both legally and in terms of social sustainability—that we examine in this article. Transfer of older collections into biobanks, or considering them as biobanks, has taken place in many countries. However, in Finland, this has created ambiguity in relation to how biobank practices are publicly presented and what collections can be transferred into biobanks in the first place. The case of legacy samples also brings forth the legislative choices Finland has made. We argue that even though European regulative framework puts high emphasis on individual rights, autonomy, and informed consent, Finnish biobank operations are in practice based on the transfer of legacy samples to biobanks, which undermines the autonomy of individuals, and the usage of samples is carried out for the most part without effectively informing their original donors.

Balancing individual autonomy and solidarity in scientific research utilizing biological materials and data has been the focus of ethico-legal discussion related to biomedical research for a long time. We believe that people’s rights to restrict consent or opt-out can be respected while building safe mechanisms to share data and promote research and development (Knoppers et al. 2014; Townend 2018; Richter et al. 2019). The new EU regulative framework applicable for processing of health-related data for research, which makes a distinction between a fundamental rights-based consent (ECHR, Oviedo Convention and EU Charter of Fundamental rights) on one hand, and legal bases for processing of personal data on the other hand (the GDPR and Clinical Trials Regulation) has further blurred the autonomy of individuals with regard to controlling the processing of their health data. We contribute to these discussions by pointing to the ambiguities and problems related to individual autonomy and to legislative weaknesses related to transferring and utilizing legacy samples in the Finnish context. In this article, we demonstrate how in the Finnish biobanks, practical operations with regard to legacy samples, and *in extensio,* for the overall majority of samples, the autonomy is reduced to the possibility of opt-out in case the donor happens to see the notice of the transfer published in the newspaper. It is likely that the donor does not have knowledge regarding the changed purpose of the diagnostic samples collected within health care (Snell and Tarkkala 2019).

In the following, we offer a socio-legal analysis (e.g., Banakar and Travers 2005) of legacy samples in Finnish biobanking, reviewing, and reflecting on the current legislation. This is done based on publicly available materials (legislation and preparatory documents) and empirical material dealing with sample collection of biobanks. In addition to the Biobank Act and its preparatory and follow-up documents, we also discuss how the current biobank law relating to transfer of legacy samples is to be regarded in light of the opinions of the Constitutional Law Committee of the Finnish Parliament. We then move on to the specific case of legacy samples, and present empirical data on the amounts of biobank samples while discussing the regulatory implications and issues raised in terms of social sustainability. The key international regulations utilized to draw attention to specific aspects of biobank operations and the Finnish Biobank Act are the European Convention of Human Rights, the Oviedo Convention, and the European Charter of Fundamental Rights as well as the GDPR and EU clinical trials regulation. In this sense, we show that a case Tupasela (2015, 380) has called “interpretive regulatory dissonance”. The concept refers to the way different countries “interpret directives and conventions in different ways”, which is seen to give rise to “vastly different legal applications and practices related to biobanking” (Tupasela 2015, 380). This is further accentuated by divergent national implementation of the GDPR rules related to scientific research posing obstacles to cross-border scientific research instead of facilitating it (for biobanking, see Slokenberga et al. 2019; for general biomedical research, see Townend 2018; Salokannel 2017). In this connection, some of the problems raised by this article relate to the fact that biobank research as defined in the current law encompasses also purely commercial drug and medical device development extending thus beyond the notion of scientific research as understood in biomedical research practice.

## Legal basis for biobank operations in Finland

### General principles of the Biobank Act

Biobank activities in Finland are defined by the Biobank Act (688/2012), which came into force in 2013. The drafting of the Biobank Act was a long process that contained disagreement about the content and scope of the law and was hampered by parliamentary elections. Initially, in 2006, a working group was set by the Ministry of Social Affairs and Health with a task to define what a biobank is and chart all the legislation that currently regulated biomedical research in Finland and elsewhere. Finally, after two rounds of comments, three drafts and over 7 years, the law came into force. However, the process has not ended, since it became apparent very quickly that there are legislative issues in need of clarification and the Act has been under reform since then.

The current Act regulates biobanks of all types, including public and private as well as clinical and epidemiological biobanks. According to the legislation, a biobank is a “unit maintained by an operator engaging in biobanking activities for the purposes of collecting and storing samples and information associated with the samples for future biobank research” [Sect. 3.1(1)].

All biobanks must apply for approval from a national ethical committee before they are registered as biobanks. In the beginning of 2014, the first two Finnish biobanks were established, registered, and started to operate. Since then, altogether, ten biobanks have been registered in the national database held by VALVIRA, the national supervisory authority for health and welfare. The biobanks in Finland are comprised of the national population-based biobank hosted by the National Institute of Health and Welfare, six biobanks operated by hospital districts, one disease-based biobank, the biobank of the national blood service, and one operated by a private health care company.

Biobank research itself is defined in the law as “research utilising the samples contained in a biobank or information associated with them for the purposes of promoting health, understanding the mechanisms of disease or developing the products and treatment practices used in health care and medical care” [Sect. 3.1(8)]. In other words, *biobank research is to be understood more broadly than mere scientific research, which also encompasses product development for general health and medical care purposes* (HE 86/2011, p. 41). “Biobank sample” is also broadly defined as consisting *both of the sample as such and any information derived from it, such as genetic information.*

A main principle of the Act (688/2012) is that informed consent of the donor is sought for the inclusion of samples in biobanks and their further use (Sect. 11 of the Biobank Act). The consent model has been promoted by biobank operators, the Ministry of Social Affairs and Health and at the policy level as one of the more positive aspects of the Act as it is regarded to enable a wide range of research, enhance the rights of the donor, and make recontacting of donors possible (HE 86/2011; Carpén and Helander 2017; Soini 2016). According to the law, the broad biobank consent also provides for adding complementary information about, for example, donor’s disease history to the biobank for the biobank to match this sort of sensitive information with the donor’s personal data (Sect. 14 Biobank Act; Opinion of the Constitutional Law Committee 10/2012). In practice, consenting to adding additional register data to the biobank data is included in the information leaflet and no separate consent is asked from the donor. The preparatory documents of the law did, however, indicate that such separate consent should have been acquired (HE 86/2011).

Moreover, the Act states that the donor has a right to limit the consent, but the current consent forms do not give the option to restrict or limit the consent.^1^ It is an either-or choice for the donor, even though the GDPR emphasises data subjects’ right to give their consent only to certain areas of scientific research (Recital 33). The consent should, according to the current international regulatory framework, safeguard the autonomy and informational self-determination of the individual as guaranteed in the European Convention of Human Rights, the Oviedo Convention, and the European Charter of Fundamental Rights.

With the entering of force of the GDPR which provides strict rules for consent as the legal basis for processing personal data, the regulatory authorities in Finland pointed to the non-compatibility of the consent used in the biobank practice with the more stricter rules for consent to be recognised as a valid legal basis for processing of personal data. Most importantly, the Finnish biobank practice did not conform with the requirements of the GDPR with regard to providing the data subject with an ability to choose whether to give a broad or a more limited consent (recital 43). This was the case even while recognising the possibility to have broad consent for research activities (recital 33). An attempt to solve this problem can be found within a new Finnish law on secondary use of social and health data (552/2019). In the transitional provisions of the law, it is stated that for the personal data collected by consent before the GDPR entered into force, the consent should be regarded as a valid legal basis for further processing presupposing that further processing relates to the same use as that referred to in the consent. According to the preparatory documents of the law, this provision is catered, in particular, towards biobank consents (HE 159/2017).

## Legacy samples and data in Finnish biobanks

The so-called legacy samples—samples collected prior to biobank legislation—constitute the basis for the Finnish biobank operations. These samples—collected for diagnostic purposes in public hospitals or for research purposes by the Finnish Institute of Health and Welfare—were identified as forming an important resource for biomedical research already over a decade ago (see STM 2007). When the process to prepare a biobank legislation in Finland was initiated, it was highlighted that Finland had already collected samples from approximately 2 million people (STM 2007, 20), and these samples should be made available for further utilization through biobanks. This was accomplished by providing in the law for the possibility to transfer most old clinical collections as well as epidemiological research cohorts into new biobanks established according to the law.

There are two types of biological samples and related data which have been transferred retroactively by virtue of the law to Finnish biobanks: diagnostic samples and samples from research projects. These data form the majority of biological materials in Finnish biobanks. The biobank law permits

- transferring of diagnostic samples which have been collected in connection with health care prior to entering of into force of the biobank law in 2013 and for which no consent exists^2^ or
- transfer of samples for such research projects which have started prior to the entering into force.^3^.

The actual transfer of legacy samples into biobanks has required permission from the regional ethical committee of the hospital district, where the samples are stored as well as from the supervisory health authority. According to the biobank law, the patients should be informed of the changed purpose related to the transfer of their samples to the biobank and of the option to prohibit it. However, in general, it has not been the custom of biobanks to re-contact patients or research participants. Instead, the possibility provided in the law to publish a notice at the website of the biobank and some daily newspapers before the transfer has been considered sufficient. In the intermediary report by the steering group of the Biobank Act (STM 2015), it was acknowledged that the notification procedure does not comply with individuals’ informational self-determination rights and that steps should be made to reinforce individual autonomy. In practice, the situation remains the same.

The law also provides for the possibility to match related register data from official registers and patient records to these retroactively transferred samples. It has not been defined in the law what such data could be. The Finnish data protection authority and the supervisory authority have stressed that these data should be closely related to the specific sample (Valvira 2017).

Moreover, when biobank operations in Finland started, it was thought that older sample collections would be transferred into biobanks within a 5-year period. The provision of the law regarding the legacy samples was originally applied to clinical collections and research projects that had started before September 2013, and these transfers were to take place by January 2018 (HE 86/2011, 72). However, also quite recently, even in 2018, acquired materials have been transferred by virtue of this provision without consent from the donors, and usually without personally informing them about the transfers.^4^ From the National Institute of Health and Welfare biobank’s webpage, for instance, it can be noted that throughout 2018, transfers of older research collections continued to take place (THL 2019b). As noted above, the Biobank Act is in the process of revision. In the 2018 draft version for a new act, it was suggested that these transfers could continue. Furthermore, the special procedure allowing the transfers could be extended to cover even sample collections that were started after the enforcement of the current Biobank Act from 2013 (Draft for Biobank Act 2018, 73).

In terms of the Finnish Constitution, the large amount of samples and attached data transferred without explicitly informing the data subjects based on an exception to the law seems problematic. As we shall demonstrate in this article, the transfers of this magnitude form in reality the basis for the biobanking operations in Finland in contrast to what the pronounced intention of the law was. According to the Constitutional Law Committee, processing of personal data, which is based on an exception to the main rule (consent in this case), cannot become the main processing activity. The biobank law is also problematic when regarded in relation to the information obligations imposed by the Oviedo Convention and, in particular, the GDPR, as we shall demonstrate later in this article.

## Current status of legacy samples in Finnish biobank operations

Currently, there are over 10 million legacy samples in Finnish biobanks. In practice, these samples and data form the majority of biological materials in Finnish biobanks (see Table 1). As the samples had been collected prior to the date, the legislation was put into force, no biobank consents as defined in the law exist for these samples. This applies both to the samples collected for diagnostic purposes in health care and samples collected for research projects that started before the law was in force. A majority of the research samples were collected with consents to the original research. This means that in practice, the foundation of the operations of the Finnish biobanking infrastructure is based on these legacy samples rather than on samples for which an explicit biobank consent has been obtained. In fact, there are nearly 100 times more legacy samples than new samples (see Table 1). Examples of such transferred legacy collections include the pathology archives of hospital districts. In Tampere, the biobank announced on 15th September 2017 a notification related to the transfer of samples taken for diagnostic purposes between 1963 and 2013. In this case, the transfer concerned 1,800,000 samples. Similarly, the Finnish Maternity Cohort (FMC), a collection of 2 million serum samples, was transferred into Borealis Biobank in Oulu in 2017.

**Table 1:** Estimated number of samples and biobank consents in six clinical biobanks in Finland

Type	Amount	
Retrospective samples		
Diagnostic samples transferred	9,950,000	
Diagnostic samples on individuals	2,900,000	
Research samples transferred	980,000	
Research samples on individuals	955,000	
Prospective consents and samples		
Biobank consents by the end of 2017	125,000	Data from all ten biobanks
Consented samples by the end of 2018	108,000	

Numbers are based partly on the e-mail responses we got from each biobank. Some biobanks provided exact numbers, while others only rough estimates. If the biobank did not respond to our queries, we have used figures that are from their web pages or presented in public presentations, and the numbers presented in a report by Medaffcon (2018)

Six biobanks are clinical biobanks operated by hospital districts that have old diagnostic sample collections and research sample collections. The four biobanks left out do not have or have not transferred diagnostic collections. THL biobank of the National Institute of Health and Welfare has transferred research samples concerning 160,000 individuals

## Mixed set of samples acquired on various legal bases: the case of the Finnish maternity cohort

One of the more illustrative examples of retrospective transfer of legacy samples into a newly formed biobank concerns the Finnish Maternity Cohort (FMC)—a collection of serum samples drawn during the first and early second trimesters of pregnancy for the screening of HIV, syphilis, and hepatitis. These samples have been gathered as a routine practice in prenatal care and stored to the Institute of Health and Welfare since 1983. The collection consists of samples from 98% of pregnant women between 1983 and 2016—almost 1,000,000 women. It should be noted that samples have been collected from nearly all women who have given birth in Finland during that period, including from those who are not Finnish nationals or who do not reside in the country anymore. The samples were gathered for a diagnostic purpose, but were stored as a research collection based on the legal duty of the Institute of Health and Welfare. Only since 2001 has there been a requirement to ask for consent for research use of the samples. In 2017, the FMC was transferred to Borealis Biobank in accordance with Sect. 13 of the Biobank Act. The transfer was announced in newspapers and web pages of National Institute of Health and Welfare (THL 2017), where it was also stated that women can opt-out from the retrospective transfer of their samples to the biobank. Since the transfer, the biobank has started to ask for a separate consent for each sample to be donated to the FMC, as required by the law. However, in 2019, the Institute of Health and Welfare webpage only provides information that the collection has been transferred to Borealis, with no further details of opt-out possibilities or about the collection given.^5^ There is only a link to the Borealis site with a short description of the cohort. The text on the page tells how to contact the biobank in case people are uncertain whether their samples are in the biobank, but there is no mention of a possibility to opt-out.^6^

The consent practices and the transfer of these legacy samples demonstrate the many difficulties we associate with Finnish legislation and the rights of individuals. First of all, the legal status of the early samples is unclear, because the boundary between a research sample and a diagnostic sample is ambiguous in this case. The samples have been used for diagnostics and were framed for the pregnant women as part of routine maternity care, but have been reassigned as a legacy collection of research samples through the establishment of the FMC. The collection now includes samples stored and utilized on three different grounds: the majority are legacy samples without any consent; the second part consists of legacy samples that have been consented to, but the transfer to biobank has been done on legal grounds, and the smallest part consists of samples collected with informed consent.

Similar problems are faced by other biobanks and countries. Biobank Graz in Southern Austria serves as a good comparison to Finnish biobanks and the transfer of older collections. The biobank was established in 2007, and since then, the already existing pathology collection of the Institute of Pathology as well as clinical collections within the Medical University of Graz have been transferred to the biobank with oldest samples dating back to 1984 (Huppertz et al. 2016, 1–2). This biobank of 7.5 million samples from over 1 million people (Huppertz et al. 2016, 1, 4) has also needed to adapt to requirements and recommendations concerning the usage of legacy samples (see, e.g., Federal Chancellery of the Republic of Austria, 2007). For example, part of the legacy samples in Graz can be used only as anonymized, since their transfer took place before clarifications for the usage of legacy samples by the national bioethics committee and was published (Huppertz et al. 2016, 4). Despite the shared challenge of the bases on which the legacy collections can be put in use, there are differences as well. In Graz, the biobank can only link indirect person-related clinical data with the samples. The direct person-related data are linked with the sample by university-based and biobank independent custodian. Moreover, the ethical approval of the biobank is renewed annually to “assure that ethical guidelines are followed any time” (Huppertz et al. 2016, 3). Such safeguards are not in place in Finland.

In the following, we analyse specific issues relating to Finnish biobank operations, the Biobank Act, and their relations to the EU General Data Protection Regulation and the related European human rights and fundamental rights framework, in particular, as they relate to informing data subjects of the use of their data. We will discuss the GDPR only and not the data protection directive 95/46/EC, although the information obligations were largely similar also under the Directive 95/46/EC. First, we analyse how the principle of purpose limitation is being overridden with regard to legacy sample collections. This highlights the fact that the legislation has not been able to guarantee a legally precise or coherent framework for biobank operations, but the purposes and the principles behind the law as stated in the preparatory documents have not been followed in practice.

## Right to privacy and data protection according to the European rights framework

According to the Article 8 of the European Convention on Human Rights and Fundamental Freedoms (ETS 005), hereinafter ECHR, everyone has the right to respect for his private and family life. The convention prohibits any interference by a public authority with the exercise of this right except such

- as is in accordance with the law;
- is necessary in a democratic society in the interests of national security, public safety or the economic well-being of the country, for the prevention of disorder or crime, for the protection of health or morals, or for the protection of the rights and freedoms of others.

Furthermore, the convention provides that the exercise of freedom of expression may be restricted for preventing the disclosure of information received in confidence (Art. 10.2). This is relevant in so far that health information is subject to strict confidentiality according to the Finnish Act on access to official documents (Law 621/99) and according to the Act on patients’ status and rights (Law 785/92). In consequence, patients expect that their health information remains confidential within the health care sector.

The Oviedo Convention,^7^ which is complemented by a number of protocols in which its principles are further elaborated and complemented with regard to specific subjects, further reaffirms the application of the fundamental human rights in relation to the integrity of persons in the field of biomedicine. It sets the interests and welfare of the human being over the sole interest of society or science (Art. 2). This means that when interpreting the convention, the interests of the individual person shall prevail over the interests of the society and research.^8^

According to the convention, any intervention in the field of medicine must be based on informed and freely given consent by the patient (Art. 5). With respect to scientific research, it is required that such consent is expressly given and that it is specific and documented. The research subjects must be informed beforehand of their rights and the safeguards provided by law for their protection (Art. 15 and 16).

The Convention also provides that the right to respect of private life extends to the information relating to person’s health. The Convention further confirms that everyone is entitled to know any information collected about her health. This is subject to person’s wish not to be informed. In addition, in exceptional cases and in the interest of the patient, the law may restrict person’s right to know about health (Art. 10). This article affirms the right to privacy of the patient as well as the right to informational self-determination over the data related to her health. The article is based on the Article 8 of the European Human Rights Convention and more broadly also on the Council of Europe Convention for the protection of individuals with regard to the processing of personal data.^9^ Finland has ratified both these conventions as well as the Oviedo Convention.

*The enforcement of the Oviedo Convention* relies on the provisions of the ECHR (Art. 32) and, consequently, on the European Court of Human Rights. While contracting parties commit themselves to insert in their national legislations necessary measures to give effect to the provisions of the Oviedo Convention and its protocols, it does not as such provide an enforcement mechanism in case member states overlook its provisions in their national laws. However, the European Court of Human Rights has the possibility to take into consideration the principles adopted in the Oviedo Convention in its judgments.^10^ To do that, this requires a violation of the rights provided for in the ECHR. In addition, the Court may issue advisory opinions on legal questions relating to the interpretation of the Oviedo Convention on request of the government of a member state or on request by the Committee setup according to Art. 32 Oviedo Convention according to the procedures provided for in Article 29, Oviedo Convention.

The EU Charter of Fundamental rights confirms the principles of the human rights conventions. The right to human dignity forms the core of human rights protection and is inviolable (Art.1). Right to the respect of the integrity of the person means that everyone has the right to respect for her physical and mental integrity. In the field of medicine and biology, this means in particular that the free and informed consent of the person concerned according to the procedures laid down by law must be respected [Art 3.2(a)]. Right to privacy is guaranteed in the Article 7 of the Charter and right to data protection in article 8. Processing of health-related data relates to all of these rights. Article 8 of the Charter constitutes the essential content of the right to data protection which may be restricted only when the requirements of Article 52.1 of the Charter are fulfilled. This article must be interpreted in light of the case law of the European Court of Human Rights.^11^

In Finland right to privacy is guaranteed in the Article 10.1 of the Finnish Constitution in which it is provided that more specific legislation relating to data protection shall be provided by law. With regard to protection of health-related data, the Constitutional Law Committee of the Finnish Parliament has adopted a strict interpretation and considers the processing of health and other sensitive data as belonging to the core of data protection (cf. PeVL 37/2013 vp). This further restricts the legislator insofar that it always has to take into account of the right to privacy as conferred under the Finnish Constitution and in the European Constitutional context (see, e.g., the opinions of the Constitutional Law Committee PeVL 13/2016 vp, s. 3—4 PeVL 1/2018, s. 3).

This means that when considering the legal framework in regard to the processing of health data, including genetic data, of the individual, it is not sufficient to take into account of only the GDPR and complementary national legislation, but also the international human rights conventions, including the Oviedo Convention as well as the Charter of Fundamental Rights and other related EU legislation such as the Clinical Trials Regulation [Regulation (EU) No: 536/2014].

## Right to information of the data subject

Right to information forms the very basis of the European privacy and data protection regulatory framework. With regard to data protection, it should safeguard the transparency of the processing. The data subject must be in a position to know when his or her personal data are being processed to enforce her rights according to the law.^12^ According to the transparency guidelines of the WP29,^13^ data subjects should be able to determine in advance what the scope and consequences of the processing are and should not be taken by surprise at a later point about the ways in which their personal data have been used. Thus, the transparency of the processing can be characterised as forming the underlying basis for any exercise of the rights of the data subject. As stated by Advocate General Cruz Villalon (9 July 2015),^14^ “the requirement to inform the data subjects about the processing of their personal data, which guarantees transparency of all processing, is all the more important, since it affects the exercise of their right of access to the data being processed and their right to object to the processing of those data, as set out in Article 14 of Directive 95/46.” This also makes it clear that the information obligation was of fundamental importance already under Directive 95/46. In this sense, transparency of the processing is the basis for guaranteeing the autonomy and informational self-determination of the individual.

In a similar line with regard to biomedical research, the Protocol on Biomedical research to the Oviedo Convention requires for a consent to be regarded as valid when the donor has been informed of all his or her rights relating to the research and of the safeguards offered by the law.^15^ When the transfer has been carried out without explicit consent and with the notification only, it has been questioned whether the donor can be considered informed (Snell and Tarkkala 2019; cf. Caulfield and Murdoch 2017). The protocol provides for detailed stipulations with regard to the information to be submitted to the research subject, while the GDPR as we shall show later is even stricter with its mandatory provisions on informing data subjects.

The information obligation portion of the Protocol to the Oviedo Convention explicitly covers information relating to the further uses of data obtained from research. The protocol expressly provides for the submission of information for any foreseen potential further uses, including commercial uses, of the research results, data or biological materials. This potential commercial use is provided for in the Finnish Biobank Act and the donors should be informed accordingly. In respect to highlighting the importance of informing subjects whether the further uses of the results, data or biological materials are part of commercial product development, the explanatory memorandum refers to the recital 26 of Directive 98/44/EC, which states that

“if an invention is based on biological material of human origin or if it uses such material, where a patent application is filed, the person from whose body the material is taken must have had an opportunity of expressing free and informed consent thereto, in accordance with national law”.^16^

This refers to the availability of research results and the eventual IPR-related arrangements related to the research project. The protocol explicitly states that the information given to the data subjects must also contain information about the source of research funding.

While information leaflets of the broad biobank consents do mention commercial product development with local and international partners, the general announcements in local newspapers relating to the transfer of old epidemiological collections do not necessarily indicate that. In addition, the content of the newspaper announcement is often hard to verify in retrospect with regard to many of the transfers, since the public announcements are not always permanently available on the webpages of the biobanks.

Another important point in the Oviedo Convention in the context of biobanking relates to biological material removed in connection with clinical intervention. The explanatory report of the Protocol on Biomedical research states that if there is an intention to utilize biological materials or personal data obtained during a medical intervention for research purposes after the medical intervention, *it is good practice to obtain a specific consent for such research uses not related to the medical intervention*.^17^ This principle is also confirmed in the EU Clinical Trials Regulation (No. 536/2014 EU), which explicitly requires a separate consent for subsequent research use of trial data.^18^ Thus, these principles are elementary both for the legacy samples based on clinical collections of hospitals, and the ones based on research projects. This principle is confirmed at the regulatory level in EU clinical trials regulation, *which distinguishes between the consent for participating in the clinical trial on one hand, and consent relating to further research uses of data derived from such trial on the other hand*. These consents would, according to the guidelines of the European Data Protection Board, be based on the international human rights and EU fundamental rights frameworks and the Clinical Trials Regulation, whereas the legal basis for the actual processing of the personal data would be based on Articles 6 and 9 of the GDPR.^19^

## Right to information of data subjects relating to transfer of legacy samples to biobanks in terms of the Finnish biobank law

The transfer of the legacy collections to biobanks entails a change of purpose for samples originally collected for diagnostic purposes. In addition, for larger research cohorts, the purpose is being changed to the extent that biobank research according to the law also encompasses commercial product development. Transfers of these collections have occurred by announcing them in major newspapers. While only intended as a last option, this has become that the main procedure biobanks have chosen to use. This is rather surprising, given that the hospitals have the contact information of their patients at hand. This is even more surprising relating to transfer of samples from longitudinal studies, since in these studies, the patients are often contacted periodically. The biobanks also attach other personal information relating to the individual from these very registers.

The preparatory documents of the biobank law (HE 86/2011) indicate that the underlying purpose of the law is to inform the data subjects of the change of purpose of the diagnostic samples and their further use. It is even stated that since the hospital districts are already in possession of the contact information of these patients, it would be easy to get in touch with them by electronic means. The information is also available in the national population register. Clearly, in light of the preparatory documents, the controller was expected to inform the data subjects individually when retroactively transferring biological materials from hospital collections to a biobank (HE 86/2011, 53). Not contacting the data subjects whose contact information was available for the data controllers would also run counter to the information requirements of Article 13 GDPR.

Data subject’s right to know about the eventual transfer of their diagnostic samples for further use in connection with biobank operations was not, however, included in the text of the law. Rather, on the contrary, the biobank law provides for the option to derogate from personally informing the data subjects in case the age or large number of the samples, or some other similar reason, which makes it burdensome to obtain the contact information of a registered individual with a reasonable effort. In this case, the notification must be published in an official paper, in a public communication network or, as necessary, in one or more daily papers (Sect. 13.4 Biobank Act). This also requires the approval of the Supervisory Health Authority. In practice, the notices have been published in a daily newspaper and they have been put up on the webpage of the biobank either permanently or for some months, after which they have been removed. Practices in this respect vary between biobanks.

There are numerous reasons why the sufficiency of notification procedure can be put under scrutiny. In terms of Article 13 of the GDPR, the original controller of biological materials of hospital collections should inform the data subjects about the changed purpose of these materials and of their right to prohibit their transfer to the biobank. Since the original controller of these materials is the hospital district, it should inform the data subjects of the changed purpose of these materials and of their possibility to prohibit the said transfer to the biobank. They should also be given information about the eventual further processing of personal data before any such further processing takes place. Furthermore, with regard to retroactively transferred samples from research cohorts in which there is no changed purpose in relation to scientific research, there is a changed purpose if those samples or the data are used for product development or other types of innovation purposes (STM 2015).

The GDPR also requires biobanks to inform data subjects in case the personal data would be transferred to a recipient in a country outside of the EEA or international organisation. These requirements are the same irrespective of whether the data are obtained directly from the data subject or from other sources.

The possibilities for making exceptions to the requirements relating to informing the data subjects differ in relation to whether the data are obtained directly from the data subject or from some other source. If the data have been obtained directly from the data subject, the only permissible exception to the information requirements is that the data subject already has all the information. Even in this case, over time, supplementary information has to be provided subject to changed circumstances. Thus, the GDPR has strict information obligations for the processing of personal data and its utilization for secondary uses more widely. As we argue throughout this article, this is a challenge for biobank practice of transferring legacy collections to biobanks.

GDPR does not permit any derogations with regard to article 13 even if the data are used for scientific research. The only possibility to override the information obligation would be under Article 23 which would require that such restriction

- is performed by a legislative measure;
- respects to the essence of the fundamental rights and freedoms;
- is a necessary and proportionate measure to safeguard.

One of the interests provided for in Art 23.2 is, public health. The application of this article is limited by the Charter of Fundamental Rights, according to which the scope of guaranteed rights and any limitation on the exercise of the rights and freedoms recognised by the Charter must be provided for by law and respect the essence of those rights and freedoms. Subject to the principle of proportionality, limitations may be made only if they are necessary and genuinely meet objectives of general interest recognised by the Union or the need to protect the rights and freedoms of others. Moreover, the Charter refers to the CoE Human Rights Convention for determining the meaning and scope of those rights when they correspond to the rights provided in the Charter (Article 52 Charter of Fundamental Rights).

The European Court of Human Rights has an established case law relating to what is to be considered as necessary in a democratic society in relation to restricting the right to privacy. According to the Court, what is necessary in terms of this article may not be interpreted as meaning what is useful, reasonable, or desirable, but it refers to *pressing social need* as a prerequisite for limiting the right to privacy. Any such legislative measure has to reflect a pressing social need and be proportionate to the legitimate aim for such measure.

In its decision *Z v Finland* the European Court of Human Rights states that “In determining whether the impugned measures were “necessary in a democratic society”, the Court will consider whether, in the light of the case as a whole, the reasons adduced to justify them were relevant and sufficient and whether the measures were proportionate to the legitimate aims pursued. In this connection, the Court will take into account that the protection of personal data, not least medical data, is of fundamental importance to a person’s enjoyment of his or her right to respect for private and family life as guaranteed by Article 8 of the Convention.

Respecting the confidentiality of health data is a vital principle in the legal systems of all the Contracting Parties to the Convention. It is crucial not only to respect the sense of privacy of a patient, but also to preserve his or her confidence in the medical profession and in the health services in general. Without such protection, those in need of medical assistance may be deterred from revealing such information of a personal and intimate nature as may be necessary to receive appropriate treatment, and even from seeking such treatment, and thereby endangering their own health and in case of transmissible diseases, that of the community (Z v. FINLAND—22009/93 [1997] ECHR 10 (25 February 1997)).

This would mean that, when applied to the restriction of the right to information of data subjects, there would have to exist a pressing social need for such derogation. This is hardly the case with regard to overlooking informing the data subjects of the transfer of diagnostic samples to biobanks for various further uses.

## Conclusions regarding the current biobank operations and discussion on future challenges

The Finnish biobanking system and the Biobank Act have been promoted internationally as one of the more advanced biobanking systems in the world. The broad consent, possibility to re-contact, and willing and positively inclined population have been identified as key components of achieving success (Snell and Tarkkala 2019). We aimed to demonstrate that the complex and changing regulatory framework creates a situation of “interpretive regulatory dissonance” (Tupasela 2015), where different countries and biobanks interpret the international and EU-level regulatory framework in a different way. What is articulated in public and how the law is applied in practice can also differ drastically. We show that the core of Finnish biobanking is formed on legacy samples that are transferred to biobanks without consent of donors and without their knowledge of the transfers. That is, even though biobanks collect prospective samples with consents; in practice, biobanks operate mostly with samples that have been transferred to their collections with a notification procedure consisting of a public announcement. These approximately 10 million legacy samples, for now, form the core of the actual biobanking operations, compared to the under 200,000 samples with biobank consent.

Even if acknowledging that similar transfers have taken place elsewhere, such as in Graz, the Finnish practice can be seen as exceptional because of the way these legacy collections were and continue to be transferred into biobank collections, while at the same time, informed consent and respect of individual autonomy are advertised by biobanks and in policies as the cornerstone of Finnish biobanking (Snell 2019). The people can hardly be expected to know that their samples are being stored, which make their ability to opt-out illusory (Snell and Tarkkala 2019). If people are not aware of the transfer or do not even know that their diagnostic samples have been saved at the hospital, the opt-out offered in connection with these transfers does not have real meaning.

While the autonomy of the individual is being obfuscated with the transfer of legacy samples to biobanks, it has also been set under strain by the GDPR which provides that authorities should not base their processing on consent, but should rather have a different legal basis, that is a law or public interest, for their processing activities. This is due to the unquestionable imbalance of power between the authority and the data subject as stated in the GDPR, according to which consent should not provide a valid legal ground in a specific case, where there is a clear imbalance between the data subject and the controller, in particular, where the controller is a public authority (GDPR, Recital 43; cf. also Ruppert et al. 2017). While a sound policy in the public sector in general, this has created confusion in the biomedical research sector, where traditionally consent has been regarded as the *sine qua non* condition for the processing of personal data. The EU commission has acquired the opinion of the European Data Protection Board regarding the very issue, and according to the EDPB, we should make a distinction between a consent based on the Human and Fundamental Rights treaties and the legal basis relating to the processing of personal data according to the GDPR (EDPB Opinion 3/2019). As we have demonstrated, in the research sector, both are applicable.

We believe that consent should not be completely abandoned as the legal basis for processing of biological material and genetic data for biomedical research, because with new consent models, such as the dynamic consent, it is possible to draft the consent in a way that it respects the requirements of the GDPR.^20^ Moreover, at the international level, consent is still the prevailing basis for processing personal data for biomedical research.

If the data processing for research purposes as such is based on law, the autonomy of the individuals could, at least to a certain extent, be respected by giving them a possibility to opt-out of further processing of their data via a permanent opt-out register. Such registers exist, for example, in Denmark and Norway. Article 21 of the GDPR permits only objecting to certain types of processing in individual cases and even rules out this for scientific research in case the processing is necessary for the performance of a task carried out for reasons of public interest (Art. 21.6). An opt-out register could thus be conceived in terms of the GDPR as a safeguard for the rights and freedoms of the individual with regard to processing of health-related data and genetic data and as a complement to the consent otherwise required by the CoE Human Rights Convention and the Oviedo Convention. Moreover, it could work as a way to introduce data minimisation principles to these large governmental data collections (EDPS Opinion 10/2017).

In Finland, setting up a permanent opt-out register within the national centralised electronic health record system *Kanta* could be a solution and this was even proposed in the preparatory and follow-up documents of the law (STM 2015). However, no such register has been set up. Rather on the contrary, it has been proposed with regard to biological samples and genetic data collected within health care that they would automatically be transferred to biobanks. This demonstrates that the Finnish biobank consent model and principles for sample gathering are not fixed yet. A draft for a revised biobank law presenting these new ways of acquiring samples was presented in 2018, and a new draft is expected to be published in 2019.

As the legacy samples have been available for use, since their transfer to biobanks starting from 2014, establishing an opt-out register now does not remove data from already-concluded projects, but it would, when accompanied by effective notification procedure at the launching stage, prevent any further use of those samples in biobanking operations without the knowledge of the data subjects. Furthermore, individuals could be notified of new research projects giving thus the data subjects a possibility to object on the use of their data in a given project or all future projects. The autonomy of individuals in terms of deciding over parts of their bodies, including the data derived thereof, could thus be restored.

As there is a thrust to be competitive and create an environment enabling biomedical research and development, it does seem that the biobank law was required in Finland because of the need to amass large amounts of health data derived from biological materials for not only scientific research, but also for product development and innovation purposes. In the recent government data policy report (VNS 2018), the biobank act was depicted as “internationally successful enabling regulation”. Moreover, contrary to what we have argued here, the Biobank Act was seen to simultaneously promote both international and national biomedical research, and “autonomy and privacy of individuals” (VNS 2018, 10). In this report, the Finnish Biobank Act was viewed as based on transparency and openness. It was also believed to have raised awareness about the ways that register data are used and engage individuals to manage their own data. As we have demonstrated, the practice of Finnish biobank operations does not fully support these claims. Quite the contrary, the regulatory environment seems to require more transparency, openness, and communication as well as reform to meet the higher standards of the GDPR.

Finnish biobank operations are from a legal point of view disconnected from the text of the law. The law sets the requisites with regard to the rights of data subjects in very general terms, and underlying meanings remain buried in preparatory works. This presents a perfect example why core principles with regard to the rights and duties of different parties must be provided for in the actual text of the law, in this case the Biobank Act, to have real meaning in practice and be binding in terms of enforcement. As for the future, the current lacunas in informing the data subjects can be corrected by providing data subjects with a real possibility to know about and control the use of their biological samples and data in biomedical research. In addition to providing legal security for all the involved parties, this would also work against losing the trust of the population in biobank operations and biomedical research in general.

## Footnotes

1 The template and content of informed consent forms has changed on several occasions, which means that in practice, the informed consents gathered differ to some degree from each other even within a biobank.

2 A health care unit that, at the time of this act's entry into force, stores biological samples generated in connection with the examination and treatment of a patient (*diagnostic samples*) and patient documents associated with such samples may transfer the samples and information associated with the samples to a biobank, secrecy provisions notwithstanding. The transfer must not jeopardise the provision and implementation of patient care (Sect. 13.1).

3 Secrecy provisions notwithstanding, an institute of higher education, a research institute, a health care unit or some other unit may transfer the samples collected and analysed in connection with a study initiated prior to this act's entry into force and the information related to them to a biobank. (Sect. 13.2)

4 As an exception to this, the National Institute of Health tweeted on 20.3.2019 that it had transferred a “Helsinki Heart Study” collection from the 1980 s and 1990 s through personal notifications and by obtaining new biobank consents from the participants (THL 2019a).

5 See https://thl.fi/fi/tutkimus-ja-kehittaminen/tutkimukset-ja-hankkeet/finnish-maternity-cohort-fmc-seerumipankki (accessed 16.5.2019).

6 https://www.ppshp.fi/Tutkimus-ja-opetus/Biopankki/Kansalaisille/Pages/default.aspx (accessed 16.5.2019).

7 Convention for the protection of Human Rights and Dignity of the Human Being with regard to the Application of Biology and Medicine: Convention on Human Rights and Biomedicine, ETS 164.

8 Additional Protocol to the Convention on Human Rights and Biomedicine, concerning Biomedical Research, CETS 195, para. 21.

9 At 63, Explanatory Report to the Convention for the protection of Human Rights and Dignity of the Human Being with regard to the Application of Biology and Medicine: Convention on Human Rights and Biomedicine, 1997.

10 Seatzu (2015).

11 For the interplay of CoE Conventions and the GDPR, see also Bygrave (2017), available at https://www.idunn.no/oslo_law_review/2017/02/data_protection_by_design_and_by_default_deciphering_the_.

12 Bygrave (2017).

13 WP29 was an advisory body that preceded the European Data Protection Board before the enactment of the GDPR.

14 Opinion of Advocate General Cruz Villalon (9 July 2015) in the Bara case (Case C-201/14) referring to application of Art. 12 of Directive 95/46, at 74.

15 Art. 5 ja 16 Oviedo Convention and Art. 14 Protocol as well as expl. memorandum at 102. Finland has not ratified the Protocol.

16 Recital 26 of Directive 98/44/EC of the European Parliament and of the Council of 6 July 1998 on the legal protection of biotechnological inventions.

17 Explanatory Report, CETS 195, 78.

18 Art. 28.2, EU Clinical Trials Regulation EU No. 536/2014.

19 Opinion 3/2019 concerning the Questions and Answers on the interplay between the Clinical Trials Regulation (CTR) and the General Data Protection regulation (GDPR) (art. 70.1.b), 23 Jan. 2019.

20 For the discussion related to dynamic consent models see, e.g. Budin-Ljøsne et al. (2017) and in particular for the UK situation Mourby et al. (2019).
